# Sarcoidosis presenting as isolated massive splenomegaly: A case report

**DOI:** 10.1002/ccr3.9283

**Published:** 2024-08-06

**Authors:** Melissa Kyriakos Saad, Imad El Hajj, Elias Saikaly

**Affiliations:** ^1^ Saint George Hospital University Medical Center Beirut Lebanon; ^2^ Department of General Surgery Saint George Hospital University Medical Center, Saint George University of Beirut Beirut Lebanon

**Keywords:** isolated, splenomegaly, sarcoidosis, splenectomy, splenomegaly

## Abstract

**Key Clinical Message:**

Sarcoidosis is a systemic granulomatous disease with an unknown cause, marked by the presence of noncaseating granulomas in the affected organs. While the pulmonary interstitium is most frequently involved, the disease can affect almost any other organ system. Extrapulmonary involvement can occur with or without lung involvement, but isolated extrapulmonary involvement is a rare event. Isolated splenomegaly is very rare and presents an uncommon manifestation of sarcoidosis, its diagnoses is a challenge due to a broad differential diagnosis. Here, we present an intriguing case of a 28‐year‐old male with isolated splenic sarcoidosis.

**Abstract:**

Sarcoidosis is a systemic disease of unknown cause, marked by the presence of noncaseating granulomas in affected organs. It most frequently impacts the pulmonary interstitium, though it can also affect nearly any other organ system. This involvement can occur with or without lung involvement, but isolated extrapulmonary cases are observed in only about 10% of instances. Furthermore, isolated splenomegaly is an exceptionally rare event and an uncommon presentation of sarcoidosis, posing a significant clinical challenge due to the wide differential diagnosis. Potential differential diagnoses include hematologic cancers, primary or metastatic splenic tumors, infiltrative diseases, inflammatory conditions, and infections. We present a noteworthy case of a 28‐year‐old with isolated splenic sarcoidosis.

## 1 INTRODUCTION

Sarcoidosis is a systemic disease characterized by the formation of noncaseating granulomas in various organs, with an unknown cause.^1^ While it predominantly affects the pulmonary interstitium,^2^ it can also impact other organs outside the lungs^3^ including the skin, eyes, and abdominal organs.^2^ This extrapulmonary involvement can happen regardless of lung involvement.^4^ In fact, isolated extrapulmonary cases is reported in about 10% of patients.^5^ Furthermore, isolated splenomegaly as the sole manifestation of sarcoidosis is extremely rare^6^ and poses significant diagnostic challenge due to the wide range of differential diagnoses, which include hematologic malignancies, primary or metastatic splenic tumors, infiltrative diseases, inflammatory conditions, and infections. To the best of our knowledge, 11 cases of isolated splenic sarcoidosis have been reported in the medical literature. Herein, we report a case of 28‐year‐old male patient presenting with isolated massive splenomegaly diagnosed isolated splenic sarcoidosis.

## 2 CASE

### 2.1 Case history/examination

This is a 28‐year‐old male patient with no past medical history born and raised in Lebanon with no travel history for 23 years presenting with fatigue and night sweats for 6 months. No fever was reported by the patient, no cough, no dyspnea, no joint pain, and no skin rash. On physical examination patient was afebrile with normal vital signs. Lungs, cardiac, and neurologic exam was normal. Abdominal exam revealed a palpable spleen reaching the pelvis and bypassing the midline, no venous collaterals were noted.

## 3 METHODS

Laboratory studies revealed thrombocytopenia, liver function tests and creatinine were within normal limits. Chest X‐ray was normal, abdominal ultrasound showed splenomegaly, and no hepatomegaly. This was followed by abdominal computed tomography (CT) which revealed the presence of an enlarged spleen measuring 46 × 20 cm with multiple hypodense lesions, no hepatomegaly and no lymphadenopathy. This was followed by a normal peripheral blood smear and a normal bone marrow examination. Consequently, positron emission tomography (PET) scan was performed showing multiple hypermetabolic splenic lesions with a SUV of 15 and no abnormal uptake in any other organ or lymph nodes.

**FIGURE 1:**
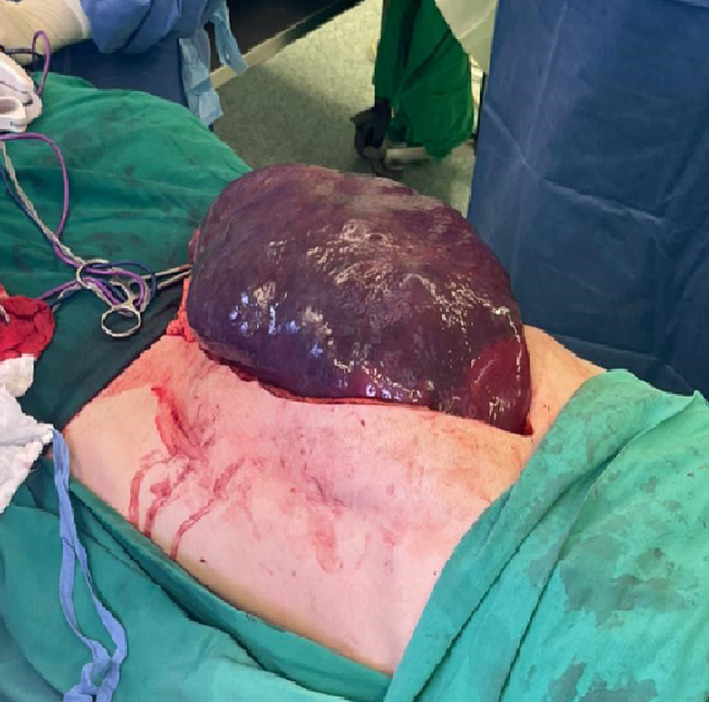
Midline laparotomy incision with spleen protruding from incision.

Consequently, open splenectomy was scheduled and done through a midline laparotomy from the xiphoid process till the pubic bone (Figure 1).

## 4 RESULTS

**FIGURE 2:**
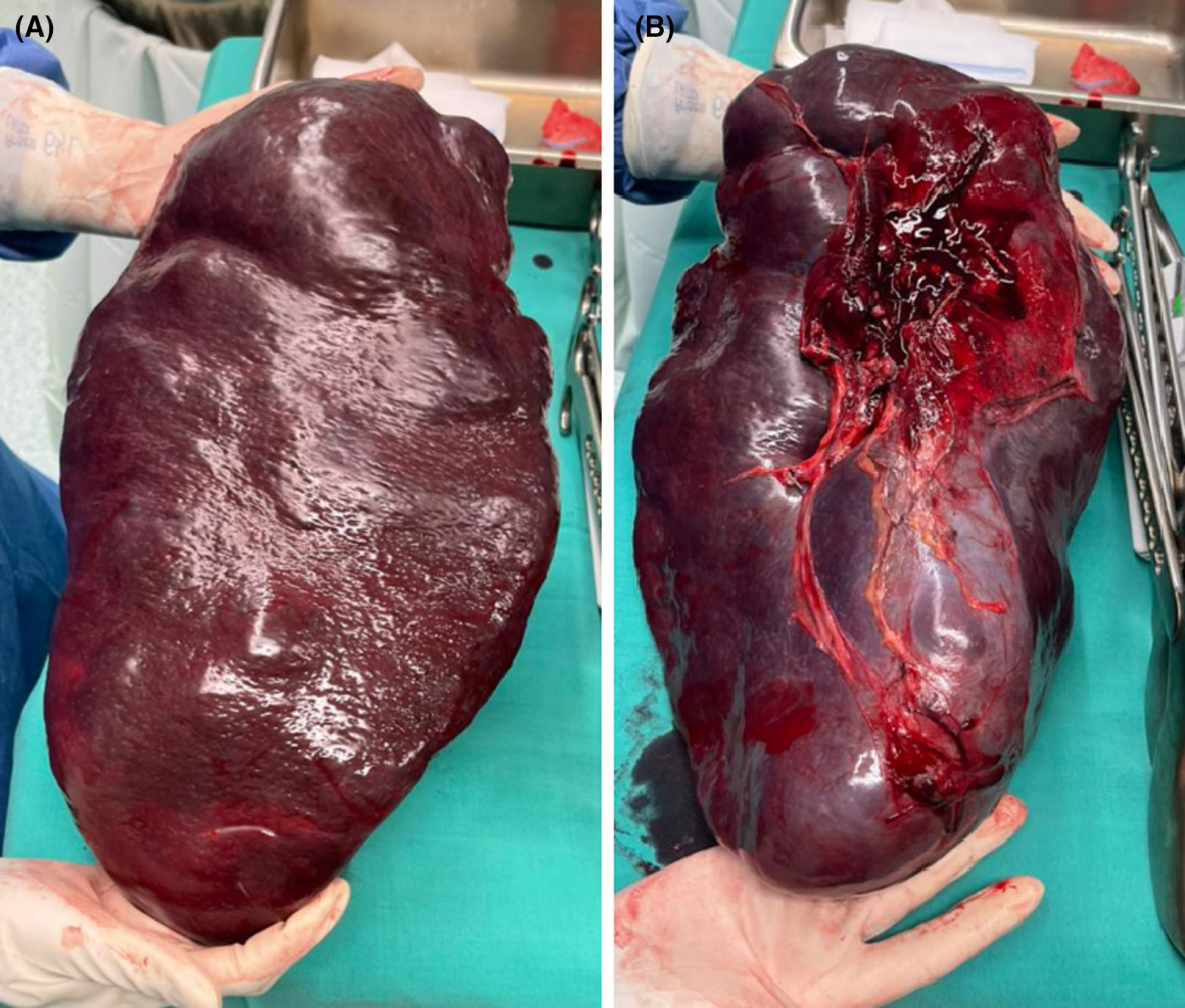
Resected specimen. (A) spleen, (B) Splenic hilum.

**FIGURE 3:**
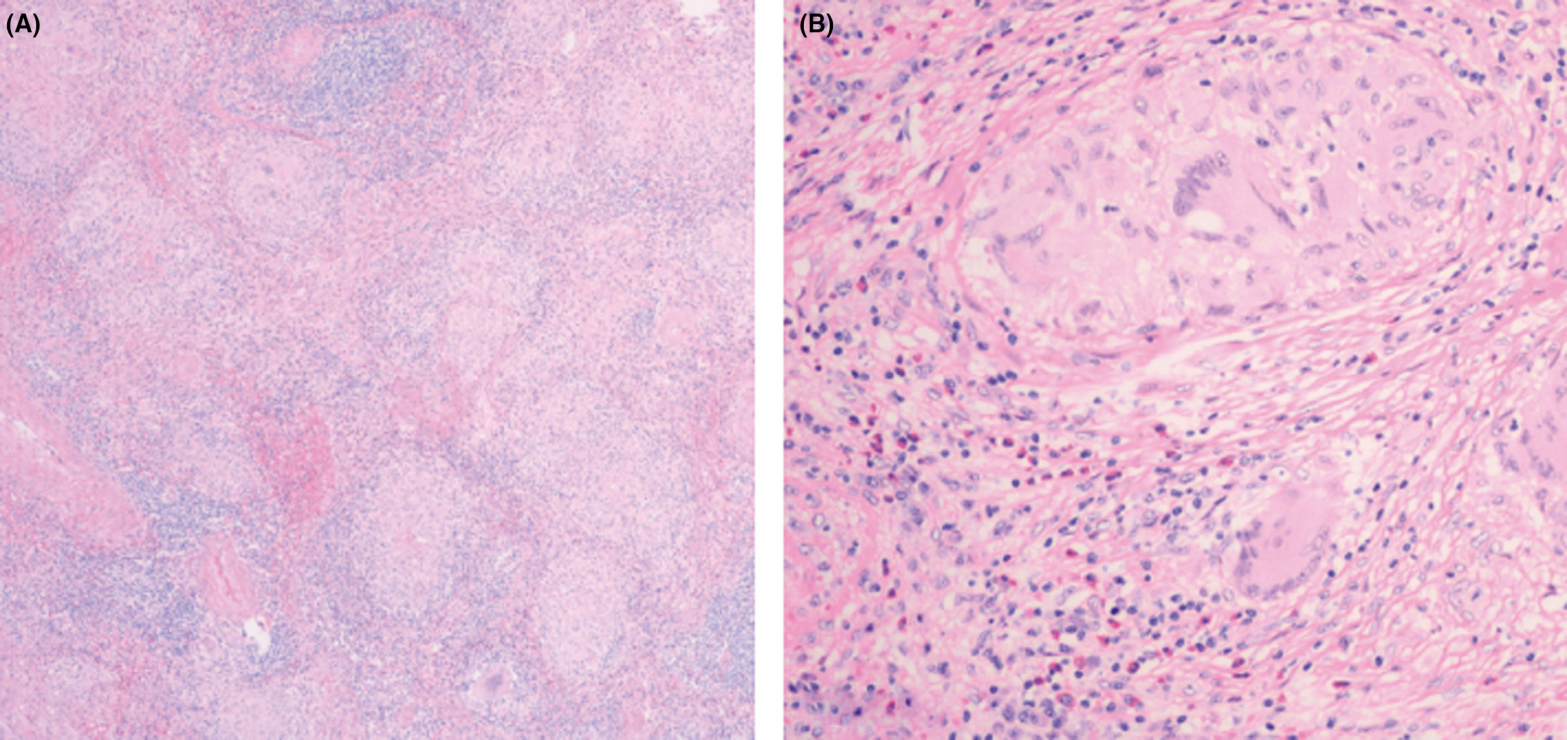
(A) Low‐power slides showing multiple noncaseating granulomas with multiple histiocyte‐consisting follicles, absence of central necrosis. (B) high‐power showing multiple noncaseating granulomas with multiple histiocyte‐consisting follicles, absence of central necrosis.

The resected spleen (Figure 2A,B) was sent for histopathologic studies. Histopathologic examination showed multiple noncaseating granulomas with multiple histiocyte‐consisting follicles (Figure 3A,B), staining for acid fast bacilli and fungus turned out to be negative.

Our patient is currently in 2 years after splenectomy and continues to be asymptomatic without any evidence of thoracic and other organ system involvement.

## 5 DISCUSSION

Isolated splenomegaly accompanied by systemic symptoms is a rare event and poses a significant clinical challenge. Differential diagnoses may include hematologic cancers, primary or metastatic splenic tumors, infiltrative disorders such as amyloidosis and Langerhans cell histiocytosis, inflammatory diseases like sarcoidosis, systemic lupus erythematosus, and rheumatoid arthritis, as well as infections like tuberculosis, fungi, and parasites. Although rare, isolated splenic sarcoidosis should be considered in patients presenting with isolated splenomegaly and systemic symptoms. Furthermore, extra thoracic involvement, especially in the liver and spleen, can occur independently of constitutional symptoms or systemic disease.^7^ Isolated splenic sarcoidosis is typically asymptomatic,^8^ although when symptoms do occur, the most common ones include abdominal pain, fever, malaise, and weight loss.^9^, ^10^ Our patient reported night sweats, and fatigue for 6 months. Amyloidosis and Langerhans cell histiocytosis in our patient were ruled out since there was no multisystem involvement. The neutrophil count in our patient was within the normal limits in blood tests done on multiple occasions making the diagnosis of splenomegaly associated with rheumatoid arthritis less likely as this often comes with neutropenia. Infectious etiology resulting in splenomegaly was ruled out by negative bacterial and fungal blood cultures, negative Acid‐Fast Bacilli (AFB) test, and negative Grocott Methenamine‐Silver Nitrate Fungus Stain (GMS) tests, as well as negative interferon tests for tuberculosis. After ruling out the above‐mentioned differential diagnosis a peripheral blood smear and bone marrow examination was done which turned out to be negative. Consequently, the patient was scheduled for a PET scan revealing multiple hypermetabolic lesions in the spleen and no abnormal uptake in other organs or lymph nodes in the body, which deemed histopathologic examination necessary for a definitive diagnosis. Given the spleen size of our patient and the risk of associated bleeding at this size and the need for histopathologic examination an open splenectomy was scheduled. Histopathologic examination showed multiple noncaseating granulomas with multiple histiocyte‐consisting follicles (Figure 3A,B), staining for acid fast bacilli and fungus turned out to be negative.

Patients diagnosed with isolated splenic sarcoidosis should also be assessed for involvement of the lungs and other organ systems both at the time of diagnosis and during follow‐up appointments. Patients with persistent systemic symptoms should be started on immunosuppressive medications such as steroids, methotrexate, and azathioprine. On the other hand, treatment is not indicated in asymptomatic patients but should be monitored closely. Those with asymptomatic, isolated splenic involvement typically have a favorable prognosis without medical therapy.^11^

Splenectomy should be considered for patients with massive splenomegaly as a preventative measure against splenic rupture, and for histopathological examination when the diagnosis is uncertain and there is a high suspicion of splenic malignancy.^12^ However, splenectomy does not alter the natural progression of sarcoidosis, and patients may still develop pulmonary or systemic sarcoidosis even after the spleen is removed.

## 6 CONCLUSION

Although rare, splenic sarcoidosis should be included in the differential diagnosis for patients presenting with systemic symptoms and splenomegaly. A histologic examination of the spleen is essential to confirm the diagnosis. Patients diagnosed with isolated splenic sarcoidosis should be assessed for thoracic and other organ system involvement at diagnosis and during follow‐up. Splenectomy should be considered in massive splenomegaly along with when histopathologic confirmation is needed.

## AUTHOR CONTRIBUTIONS

**Melissa Kyriakos Saad:** Investigation; methodology; project administration; resources; supervision; writing – original draft; writing – review and editing. **Imad El Hajj:** Investigation; methodology; resources; supervision; validation; writing – original draft. **Elias Saikaly:** Conceptualization; investigation; methodology; project administration; resources; supervision; validation; visualization; writing – original draft; writing – review and editing.

## FUNDING INFORMATION

N/A.

## CONSENT

Written informed consent was obtained from the patient to publish this report in accordance with the journal's patient consent policy.

## Data Availability

all data available upon request from corresponding author.
